# Assessing Variation in US Soybean Seed Composition (Protein and Oil)

**DOI:** 10.3389/fpls.2019.00298

**Published:** 2019-03-11

**Authors:** Yared Assefa, Larry C. Purcell, Montse Salmeron, Seth Naeve, Shaun N. Casteel, Péter Kovács, Sotirios Archontoulis, Mark Licht, Fred Below, Herman Kandel, Laura E. Lindsey, John Gaska, Shawn Conley, Charles Shapiro, John M. Orlowski, Bobby R. Golden, Gurpreet Kaur, Maninderpal Singh, Kurt Thelen, Randall Laurenz, Dan Davidson, Ignacio A. Ciampitti

**Affiliations:** ^1^Department of Agronomy, Kansas State University, Manhattan, KS, United States; ^2^Department of Crop, Soil, and Environmental Sciences, University of Arkansas, Fayetteville, AR, United States; ^3^Department of Plant and Soil Sciences, University of Kentucky, Lexington, KY, United States; ^4^Department of Agronomy and Plant Genetics, University of Minnesota, Saint Paul, MN, United States; ^5^Department of Agronomy, Purdue University, West Lafayette, IN, United States; ^6^Department of Agronomy, Horticulture & Plant Science, South Dakota State University, Brookings, SD, United States; ^7^Department of Agronomy, Iowa State University, Ames, IA, United States; ^8^Department of Crop Sciences, University of Illinois at Urbana–Champaign, Urbana, IL, United States; ^9^Department of Plant Sciences, North Dakota State University, Fargo, ND, United States; ^10^Department of Horticulture and Crop Science, The Ohio State University, Columbus, OH, United States; ^11^Department of Agronomy, University of Wisconsin–Madison, Madison, WI, United States; ^12^Department of Agronomy and Horticulture, University of Nebraska System, Lincoln, NE, United States; ^13^Delta Research and Extension Center, Mississippi State University, Stoneville, MS, United States; ^14^Department of Plant, Soil and Microbial Sciences, Michigan State University, Allegan, MI, United States; ^15^Illinois Soybean Association, Bloomington, IL, United States

**Keywords:** crop environment, soybean management, protein yield, oil concentration, seed quality

## Abstract

Soybean [*Glycine max* (L.) Merr.] seed composition and yield are a function of genetics (G), environment (E), and management (M) practices, but contribution of each factor to seed composition and yield are not well understood. The goal of this synthesis-analysis was to identify the main effects of G, E, and M factors on seed composition (protein and oil concentration) and yield. The entire dataset (13,574 data points) consisted of 21 studies conducted across the United States (US) between 2002 and 2017 with varying treatments and all reporting seed yield and composition. Environment (E), defined as site-year, was the dominant factor accounting for more than 70% of the variation for both seed composition and yield. Of the crop management factors: (i) delayed planting date decreased oil concentration by 0.007 to 0.06% per delayed week (*R*^2^∼0.70) and a 0.01 to 0.04 Mg ha^-1^ decline in seed yield per week, mainly in northern latitudes (40–45 N); (ii) crop rotation (corn-soybean) resulted in an overall positive impact for both seed composition and yield (1.60 Mg ha^-1^ positive yield difference relative to continuous soybean); and (iii) other management practices such as no-till, seed treatment, foliar nutrient application, and fungicide showed mixed results. Fertilizer N application in lower quantities (10–50 kg N ha^-1^) increased both oil and protein concentration, but seed yield was improved with rates above 100 kg N ha^-1^. At southern latitudes (30–35 N), trends of reduction in oil and increases in protein concentrations with later maturity groups (MG, from 3 to 7) was found. Continuing coordinated research is critical to advance our understanding of G × E × M interactions.

## Introduction

Soybean seed yield and quality are functions of genotype (G), M, E, and their interaction (G × E × M), but how each individual factor affects seed yield and quality, and their level of significance is not well established (Rao et al., 2002; Assefa et al., 2018). Previous studies have reported mixed results for climatic factors, such as temperature and solar radiation on soybean growth and seed composition (Cartter and Hopper, 1942; Maestri et al., 1998: Grieshop and Fahey, 2001; Dardanelli et al., 2006; Goldflus et al., 2006). For example, a few studies reported an increase in oil without any effect on protein concentration as temperature increases (Howell and Cartter, 1958; Ren et al., 2009; Mourtzinis et al., 2017a). Kumar et al. (2006) showed a positive linear relationship between temperature and protein concentration, but a negative relationship between temperature and oil concentration. Some other studies reported quadratic relationships of oil and protein concentration with temperature (Piper and Boote, 1999; Pipolo et al., 2004).

Soybean seed composition is also affected by the application of nutrients and water depending on the availability of resources and crop yield potential. Ham et al. (1975) and Nakasathien et al. (2000) reported an increase in protein concentration and a decline in oil with increased application of nitrogen (N) fertilization, whereas Wood et al. (1993) reported lack of response from both soybean oil and protein concentration to fertilizer N application. In testing the different combinations of foliar nutrient fertilization on soybean in 112 field trials in Iowa, Haq and Mallarino (2005) concluded that total oil and protein production response to fertilizer followed a similar patter with yield response. However, they noted small, erratic and inconsistent changes in oil and protein concentrations. In a meta-analysis, Rotundo and Westgate (2009), concluded that providing supplemental N increased both protein concentration and content but slightly decreased oil concentration. The impact of irrigation on protein concentration was dependent on the soybean genotypes (Boydak et al., 2002; Bellaloui and Mengistu, 2008). A decline in protein concentration with water deficit during reproductive stages was reported by Carrera et al. (2009), whereas Kumar et al. (2006) reported an increase in protein concentration with decreased precipitation. In a meta-analysis for soybean seed composition, Rotundo and Westgate (2009) documented an overall relative positive response to water stress of protein concentration, regardless of the timing of the stress and studies (field and pot trials). The same authors found an overall negative impact of water stress on both oil concentration and content.

Besides E and inputs, crop M and G also affect soybean seed composition and yield. A negative impact of continuous soybean cropping on protein and oil composition relative to soybean rotation with corn (*Zea mays* L.) (Bellaloui et al., 2010) or its intercropping with sorghum (*Sorghum bicolor* L.) (Elsheikh et al., 2009) has been noted. The impact of planting date on oil and protein concentration appears mixed with a benefit for oil from early planting, whereas late planting promoting protein concentration (Robinson et al., 2009; Bellaloui et al., 2015), or a benefit to both protein and oil from early planting (Jaureguy et al., 2013). Further analysis of impact of planting data by genotypes and possible contributing factors related to obtained results were also topics of study (Rowntree et al., 2013, 2014). The difference in the definition of early or late planting and lack of detailed weather characterization may complicate interpretation of these reports. Therefore, the objective of this manuscript was to identify the main effects of G, E, and M factors influencing seed composition (protein and oil) and their association with soybean yield through meta-analysis and synthesis of a database obtained across the United States.

## Materials and Methods

**FIGURE 1:**
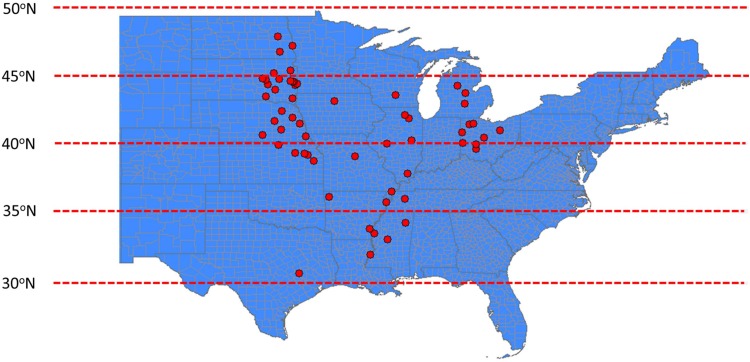
Partial map of the United States (US) showing the locations for all field trials conducted and utilized to create the soybean seed yield and quality (protein and oil) database. Geographical locations are presented as red circles.

The dataset (13,574 data points) consisted of 21 studies from 11 states within the major soybean producing regions of the United States Impacts of different treatments on soybean seed composition (protein and oil) and yield were among the main response variables (Table 1). In addition, several studies were conducted across years at the same locations. Location of each study is presented in Figure 1. Treatments and experimental designs varied across studies. However, each of these studies have reported similar independent variables or covariates (planting and harvesting date, location, year, fertilizer rate, other M) and comparable response variables (oil, protein, or yield). Analysis of the overall distribution and relationships among response variables were conducted for the entire data set but specific factor effect analysis was conducted using studies that have similar range of treatments. Soybean seed oil and protein concentrations were measured using near infrared transmittance (NIT) spectroscopy (Infratec 1241 Grain Analyzer, Foss Instruments, Eden Prairie, MN or DA 7250 NIR analyzer, Perten Instruments, Inc., Springfield, IL, United States) and seed yield measurements were adjusted to 130 g kg^-1^ seed moisture content. By creating a regional and large database, we have increased the power of detecting the effect of the treatments representing G × E × M combinations on the measured response variables.

**Table 1:** Soybean database on yield and seed quality (protein and oil) presenting for each study number of data points, United States, treatments of study (**X**), and other independent or response variables (X) measured and available.

NO.	Data point	Coordinating State	Treatments, covariates, or response variables measured																				
			
														Other		
					Dates	Seeding		Genetics		Nitrogen			nutrients			Other Management					Response		
											
			Year	Location	Planting	Rate	Spacing	Maturity	Cultivar	Rate	Source	Timing	P	S	Mn	Tillage	Seed trt.	Rhizobi.	Rotation	Foliar app.	Yield	Oil	Protein
1	6613	AR	X	X	**X**	X	X	**X**	**X**	_	_	_	_	_	_	_	_	_	_	_	X	X	X
2	120	AR	X	X	X	X	X	_	X	**X**	**_**	**_**	_	_	_	X	_	_	_	_	X	_	_
3	1583	IL	X	X	X	X	X	X	X	_	**X**	**X**	_	_	_	X	_	_	_	_	X	X	X
4	48	IA	X	X	**X**	_	_	_	X	_	_	_	_	_	_	_	_	_	_	_	X	X	X
5	756	KS	X	X	X	_	_	_	X	**X**	_	**X**	_	_	_	_	_	_	_	**X**	X	X	X
6	320	MI	X	X	_	_	_	_	_	_	_	**_**	_	_	_	_	X	_	_	_	X	X	_
7	144	MI	X	_	**X**	**X**	_	_	**X**	_	_	**_**	_	_	_	_	**X**	_	_	**X**	X	X	X
8	48	MI	X	_	_	**X**	_	_	**X**	_	_	**_**	_	_	_	_	_	_	_	_	X	X	X
9	272	MS	X	_	_	_	_	_	_	**X**	**X**	**X**	_	_	_	_	_	_	_	_	_	X	X
10	86	NE	X	X	X	X	X	_	X	**X**	_	**X**	_	_	_	X	_	_	_	_	X	X	X
11	86	NE	X	X	X	X	X	_	X	**X**	**X**	_	_	_	_	X	_	_	_	_	X	X	X
12	80	ND	X	X	X	X	X	X	X	**X**	**X**	**X**	_	_	_	**X**	**X**	_	_	_	X	X	X
13	56	ND	X	X	X	**X**	**X**	X	X	_	_	_	_	_	_	X	_	_	_	_	X	X	X
14	120	ND	X	X	X	X	X	**X**	**X**	_	_	_	_	_	_	X	_	_	_	_	X	X	X
15	108	ND	X	X	X	X	X	X	X	**X**	**X**	X	_	**X**	_	X	_	_	_	_	X	X	X
16	384	ND	X	X	X	X	X	**X**	**X**	**X**	**X**	**X**	_	_	_	**X**	_	_	_	_	X	X	X
17	384	OH	X	X	X	X	X	X	X	_	_	_	_	_	**X**	X	**X**	**X**	_	**X**	X	X	X
18	140	OH	X	X	**X**	X	X	X	X	**X**	_	_	**X**	_	_	X	_	_	_	_	X	X	X
19	280	SD	X	X	X	_	_	X	X	_	_	_	**X**	_	_	_	_	_	_	_	_	X	X
20	832	SD	X	X	**X**	**X**	X	X	X	_	_	_	_	_	_	X	**X**	_	_	_	X	X	X
21	1568	WI	X	X	_	_	_	_	**X**	_	_	_	_	_	_	**X**	**X**	**X**	**X**	_	X	X	X


### Statistical Analysis

We used a five-step process to analyze the data. First, we determined the distribution of oil, protein, and seed yield across the US soybean database. Yield distribution was further investigated by latitude groups obtained by the UNIVARIATE procedure in SAS (SAS Institute, 2012). Then, the overall relationship between oil concentration and oil yield with seed yield, protein concentration and protein yield with seed yield, and the relationship between oil and protein concentrations were all studied using PROC REG procedure in SAS. Even though the main objective was to obtain the combined overall relationship among the response variables, analysis was also conducted by study or group of studies (for locations with multiple studies).

Second, the database was classified into 103 Es based on the site-year information. A 1-year field trial with different treatments (planting date, tillage, or other) at a given location was considered as an E. The first analysis was conducted to determine the impact of the E on protein, oil, and seed yield. These three response variables were modeled separately against E as a fixed variable in the PROC GLM procedure in SAS. The result of this model fit, the R^2^, was used to explain the proportion of variation explained by or accounted for E for protein, oil, and seed yield, as response variables.

In the third step, the impact of different M factors was investigated by dividing the data into two groups: (i) planting date or (ii) other M factors. The impact of planting date, as continuous variable was studied by latitude groups. Planting dates were grouped by weeks from the earliest to the latest planting date for all the studies gathered in the database. Then, the PROC MEAN procedure was used to determine the minimum, mean, and maximum values of oil, protein, or seed yield for each planting week. Interpretation of data was done based on linear fitted trends to mean values of the seed composition and seed yield over planting week by latitude group. For M variables such as tillage, rotation, seed treatment that have a categorical contrast of treatments (e.g., for tillage factor, conventional versus no-till), a meta-type analysis was conducted. The forest-plot of the mean differences was used to present overall effects of each M factor and this analysis was conducted in R using R package meta (Lewis and Clarke, 2001; R Development Core Team, 2012). These forest plots present the difference between the mean response of improved technology over the traditional (control) and the standard error attached to the mean difference.

The fourth step considered application of inputs such as N fertilizer effects on oil, protein, and seed yield. The amount of N fertilizer varied from study-to-study. Therefore, we grouped the N rates into five categories (control, 0 kg ha^-1^, 10–50 kg ha^-1^, 50–100 kg ha^-1^, 100–150 kg ha^-1^, and >150 kg ha^-1^) and determined the exceedance probability of seed composition and yield level at each N rate category using UNIVARIATE procedure of SAS. Exceedance probability here is defined as the probability of obtaining oil, protein, or yield exceeding the indicated amount for each N rate category.

For the fifth step, the effect of MG of varieties (G) was studied by latitude groups. The minimum-, mean-, and maximum-oil, protein, or seed yield by MG of each variety by latitude was determined using the PROC MEAN procedure. Regression analysis was conducted on mean and the coefficient of determination (*R*^2^) is presented when the relationship was statistically significant (*P* < 0.05). Interpretation of data was done by studying the trends of the minimum, mean, or maximum values of the variables as MG changes in each latitude group. We recognize that this analysis does not account for genetic differences within MG.

## Results and Discussion

### Environment, Data Distribution, and Relationships

**FIGURE 2:**
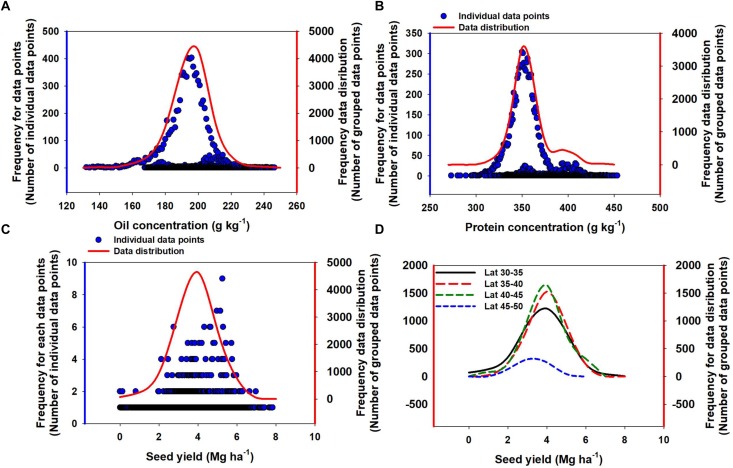
Frequency distribution for individual data points and an overall distribution for soybean oil concentration **(A)**, protein concentration **(B)**, seed yield **(C)**, and seed yield by latitude **(D)**. Line data distributions were calculated by grouping values to the nearest whole number and adding the frequency of each of the grouped values to arrive at the frequency for the group.

**FIGURE 3:**
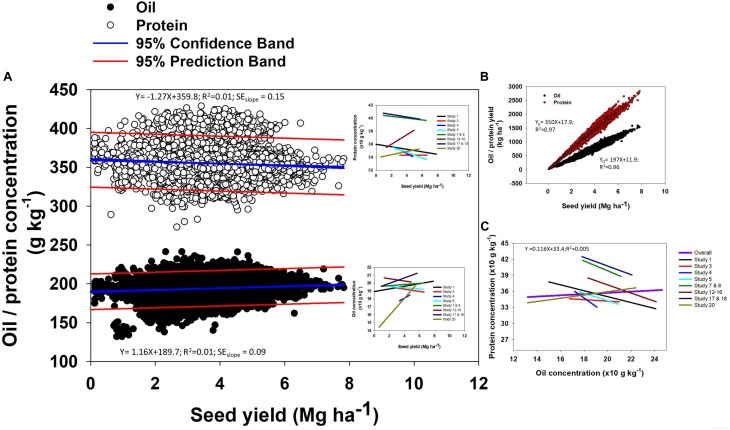
Relationships between oil and protein concentration with seed yield **(A)**, seed yield with oil and protein yield **(B)**, and protein concentration and oil concentrations **(C)**. Study numbers are in the order presented in Table 1.

Oil concentration in soybean ranged from 132 to 246 g kg^-1^ (Figure 2). The mean oil concentration was 195 g kg^-1^, and 90% of the data were within a 30 g kg^-1^ range, i.e., from 180 to 210 g kg^-1^. Protein concentration ranged from 273 to 454 g kg^-1^ (Figure 2). The mean protein concentration was 357 g kg^-1^, and 90% of the data were within a 60 g kg^-1^ range, i.e., from 330 to 390 g kg^-1^ (Figure 2). Seed yield ranged from minimum of 0.1 to maximum of 7.8 Mg ha^-1^. The mean seed yield was 3.8 Mg ha^-1^, and 90% of the data ranged from 3 to 6 Mg ha^-1^. Across different seed yield values, there was a slight change in both oil and protein concentration (Figure 3). Overall, oil concentration increased slowly at rate of 1.2 g kg^-1^ per Mg seed yield increase but protein concentration decreased at 1.3 g kg^-1^ per Mg seed yield increase. When relationships were investigated by study (as presented on insets, Figure 3A), 66% of studies resulted in a slight negative trend for protein concentration as yield increased and the other 33% showed a slightly positive relationship for protein concentration and yield. This differed from the relationship between oil concentration and yield where 63% of studies supported a positive relationship and the other 37% displayed a slightly negative relationship. However, oil and protein concentration should not be confused with oil or protein yield (production per unit area). Both oil and protein yields increased in proportion to seed yield at rates of 198 and 350 kg per Mg seed yield increase, respectively (Figure 3B), a similar relation reported from a recently published different data set (Assefa et al., 2018). From the above relationship we can calculate that there was a 1.7 kg increase in protein yield for each kg oil yield increase. Thus, the overall ratio for soybean of protein- to oil-yield was 1.7. Often, a negative correlation between oil and protein concentration was reported. When pooling data across all our Es, there was no significant relationship between oil and protein concentration (Figure 3C). However, a tendency for a negative relationship between oil and protein concentration was observed when plotting data separately for each of the studies evaluated in the database (Figure 3C).

The reason why there was a positive seed-yield with protein- and oil- yield but a negative seed-yield to protein concentration relationships by E is because concentration is a relative measure and yield is an absolute measure. When seed-yield increase by 1 kg, protein-yield increased (350 g kg^-1^; Figure 3B) less than the mean protein concentration (357 g kg^-1^; Figure 2). From the oil perspective, for the same 1 kg seed yield increase, oil-yield increased (198 g kg^-1^; Figure 3) slightly more proportionally than the mean oil concentration (195 g kg^-1^; Figure 2). The different trends for protein-yield and oil-yield relative to their mean concentrations in seed provide evidence for the negative relationship between oil and protein concentration by E. This positive relationship between protein- and oil-yield with seed yield but a decline in protein concentration by E reported in this study is in line with results presented by Ray et al. (2006) and Rotundo and Westgate (2009). From a genetic standpoint, Chung et al. (2003) reported a negative correlation between protein concentration and yield, suggesting that the energetic cost associated with increased protein deposition is energetically costly than commonly assumed. When the data is analyzed across Es (not within an E), greater protein concentration was accompanied by also high oil concentration; while in Es with low protein, oil also presented low concentrations all relative to the high protein-oil Es. Therefore, protein-to-oil concentration relationships were negative within an E but no relationship or positive relationship tendencies across Es.

**FIGURE 4:**
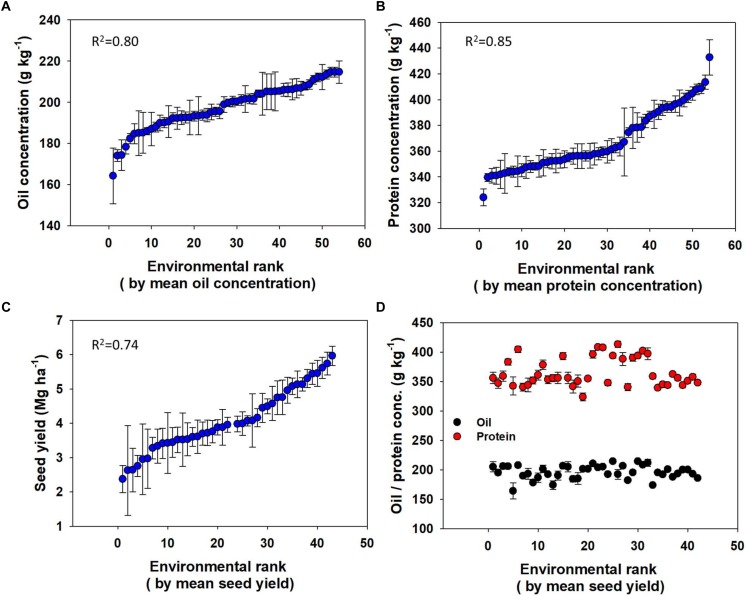
Variation in oil concentration **(A)**, protein concentration **(B)**, and seed yield **(C)** across environments each ranked with respective variables and oil and protein concentration **(D)** across Es ranked with mean seed yield.

Environment alone explained significant variation in mean oil (*R*^2^ = 0.80) and protein (*R*^2^ = 0.85) concentrations, and seed yield (*R*^2^ = 0.74; Figure 4). The difference in concentration between the lowest and highest ranked Es (Figure 4A–C) were about 50 g kg^-1^ for oil and 110 g kg^-1^ for protein. The range between the lowest and highest average yielding E was about 4 Mg ha^-1^ for seed yield. However, yield and seed composition (protein and oil) rankings were not significantly correlated; neither oil nor protein concentrations were linearly related to the yield ranking (Figure 4D).

Distribution of both oil and protein concentrations had a relatively narrow variation, primarily explained by the E (80–85%). Likewise, E accounted for a major proportion of variation (74%) for soybean seed yield. Environmental variation primarily encompasses differences in soil type and climate that affect plant growth, development, and yield formation. These results are generally in agreement with Mueller et al. (2012), highlighting the impact of weather affecting yields. Specifically, to soybean, Grieshop and Fahey (2001) reported that environmental conditions have great impact on seed composition. A synthesis analysis by Mourtzinis et al. (2018) also concluded that 68% of the variability in soybean yield in the United States was associated with variations in the E. Analysis of specific effects of environmental factors such as temperature and rainfall on seed composition and yield was not addressed in this study. Other studies have reported relationship between temperature or rainfall (water) with oil and protein content (Howell and Cartter, 1958; Piper and Boote, 1999; Pipolo et al., 2004; Kumar et al., 2006; Carrera et al., 2009; Ren et al., 2009; Rotundo and Westgate, 2009; Mourtzinis et al., 2017a). Our result, in general, suggest that Es with great seed yield have greater oil and protein yields, however, within an E oil concentration increase with yield while protein concentration decreases for possible reasons suggested above.

### Management

**FIGURE 5:**
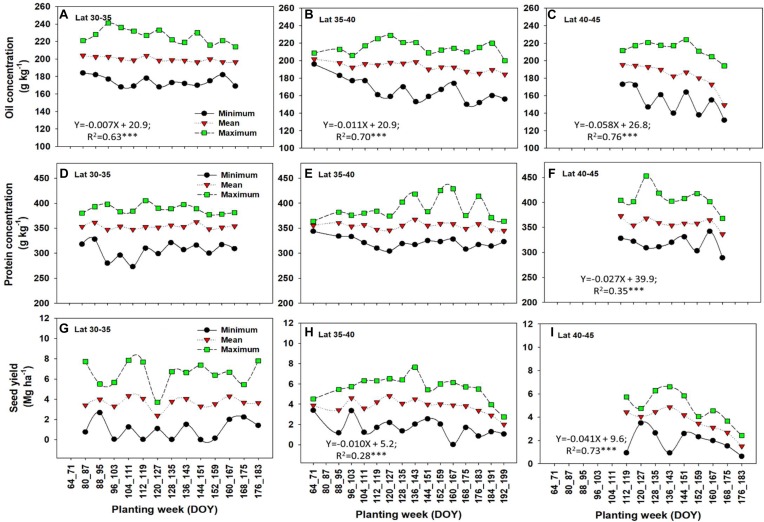
Minimum, mean, and maximum values of oil concentration **(A–C)**, protein concentration **(D–F)**, and seed yield **(G–I)** by latitude and at different planting weeks. There was no enough planting data variation for latitude >45 N. Regression equations are given for significant relation (*P* < 0.05^∗^ or 0.001^∗∗∗^) between mean response and planting week.

Planting date is an important M factor affecting the overall length of the growing season, the time and developmental stage the crop is exposed to the E, and resource availability during the cropping cycle. In a sense, the impact of planting date is connected to E and resources (nutrients, solar radiation, and water) which are vital to plant growth. Planting date did affect oil concentration and seed yield across latitudes but larger impacts were documented at northern latitudes, 40–45 N (Figure 5). In southern latitudes (30–35 N), mean oil concentration significantly declined with later planting dates at a rate of -0.007% (planting week)^-1^ (Figure 5A). In mid latitudes (35–40 N), mean oil concentration also declined significantly as planting was delayed at a rate of -0.011% (planting week)^-1^ (Figure 5B), representing a 36% larger reduction in mean oil concentration relative to the southern latitudes (30–35 N). In northern latitudes (40–45 N), a sharper, significant decline in mean oil concentration was documented as planting date was delayed at an overall rate of -0.058% (planting week) ^-1^ (Figure 5C), an 88% larger impact of planting date on oil for this latitude relative to the southern latitudes (30–35 N).

Protein concentration was not significantly affected in most of the latitude groups (Figure 5D,E), except for the northern latitude range 40–45 N, presenting a significant decline for overall mean protein concentration at a rate of -0.027% planting week^-1^ (Figure 5F). For seed yield, mean yield was not affected by planting date at the southern latitude range (30–35 N), but a significant negative impact was observed for the mid-latitude range (35–40 N) at a rate of -0.010 Mg ha^-1^ (planting week) ^-1^ (Figure 5H) and for the northern latitudes (40–45 N) at a rate of -0.041 Mg ha^-1^ (planting week) ^-1^. The latter latitude range presented a larger significant impact on yield as planting date was delayed (Figure 5I). Mean seed yield values tended to decline with planting after 145 DOY in both mid- and high-latitude ranges.

This lack of yield response to planting date in lower latitudes is in contrast with the study by Egli and Cornelius (2009), that found a rate of decline with delay in planting date of 0.7% in the Midwest, and 1.1–1.2% in the Midsouth and Deep South. Our result on lack of yield response in lower latitude also differs from the conclusions from Salmeron et al. (2014) and Salmeron et al. (2016), which are based on a large subset of data from this paper but considered planting data effect by MG and found significant yield reductions when planting date was delayed. In the analysis by Salmeron et al. (2016), yield showed a quadratic or negative linear response to day of planting depending on the location and soybean maturity. Delaying planting date from mid-May to early June decreased yields by 0.09 to 1.69% per day of delay in planting date (approximately 0.003 to 0.414 Mg ha^-1^ week^-1^) (Salmeron et al., 2016). Here, we looked at the main effect of planting date (not interaction with MG) and the lack of response to planting date in the lower latitudes perhaps is result of averaging effect over many MGs that responded differently at different planting windows.

In summary, late planting date negatively impacted oil concentration and seed yield. The impact of planting date on oil or seed yield, was moderate at the southern latitude (30–35 N) compared to the northern latitudes (40–45 N). Protein concentration was only significantly affected by late planting date for the northern-latitude range (40–45 N). In southern latitudes, there is a wider “window” for planting due to longer frost free time period and generally preferred growing condition. Significant decline in oil concentration but not protein concentration did not mean protein concentration is not negatively affected. Since concentration is a relative measure, if oil content increases faster than protein content when yield increase, then when seed yield decreases due to delayed planting date, oil content should be potentially decreasing faster than protein content and therefore the decrease in oil is more significant than protein concentration. Planting dates are important in northern latitudes due to the shorter growing season, and a similar conclusion has been documented for corn planting date range across latitudes in the United States (Long et al., 2017). The importance of planting date to soybean seed composition and yield was reported (Jaureguy et al., 2013; Bellaloui et al., 2015; Mourtzinis et al., 2017a) but with conflicting results. The unique aspect of this study regarding planting date is that our report covers trends with data from multiple sites across latitudes and planting weeks within each latitude. In a structured planting date studies, results are presented by E and relative to early and late scheduled planting dates in each study. In most planting date studies, what is early and late planting is subjective and usually defined in relative to planting dates of the study each year. Within a planting date study, what is early and what is late differs by year to the extent that the late planting date 1 year may become early planting date in another year. The advantage of this meta-analysis is the ability to detect the overall trend across studies regardless of year-to-year variability. Our limitation is in dissecting planting date effect by other interacting factors such as varieties or maturity, which vary by studies included in the analysis.

**FIGURE 6:**
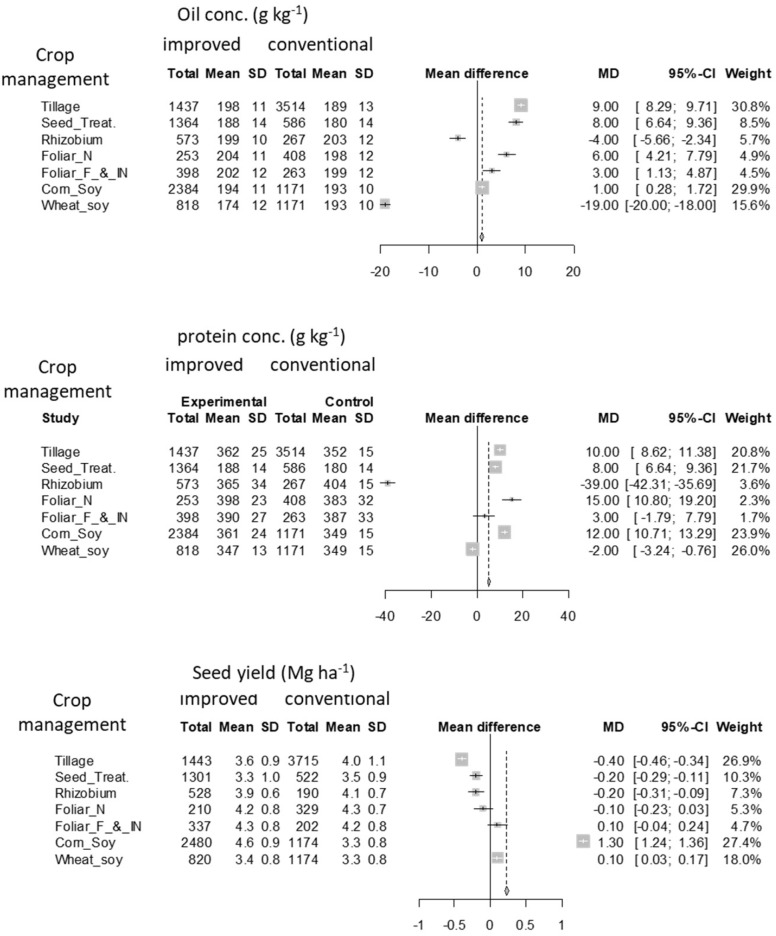
A detailed forest plot for the effect of improved tillage, seed treatment, rhizobium inoculation, foliar N application, foliar fungicide and insecticide application, corn-soybean, and spring wheat-soybean double crop rotation over conventional approaches on oil concentration, protein concentration, and seed yield. Light symbols with square box in mean difference represent factors with greater weight and black dot with a standard error bar represent factors with less weight. The weights of each factor and dotted line in mean difference were important points to discuss if an overall factor effect and comparison of factors was the objective, however, the intention of the current analysis is only to present the individual management (M) factor effect.

Crop management factors such as no-till, seed treatments, foliar N, fungicide and insecticide applications, and rotation, by improving crop growing conditions through conserving or suppling water and nutrients or improving soil physicochemical conditions and protecting the crop from disease, had an overall positive effect on both oil and protein concentrations (Figure 6). Some of these M factors (no-till, seed treatment, foliar N) which showed a positive impact on seed composition did not necessarily affect yields relative to their conventional production techniques. Overall seed yield did not seem to benefit from most of these improved crop M systems except for crop rotation and foliar fungicide and insecticide applications. Similar conclusions on a positive impact of rotation and no effect from seed treatment were reported by Mourtzinis et al. (2017b). The positive impact of diverse rotation for seed composition and yield is evident for soybean and other crops (Riedell et al., 2009; Bellaloui et al., 2014). A divergence in soil microbial population between mono-cropping and rotation was among the main mechanisms for rotation positively affecting crop growth, yield, and seed composition in oil seed rape (*Brassica napus;* Hilton et al., 2013). A mixed response to tillage treatments by year of experiment was reported by Singer et al. (2008). Research on the effect of M factors such as tillage on seed composition is limited. This analysis calls for the need of investigations on the most relevant M factors and mechanisms impacting seed composition to better understand the interaction between G × E × M.

### Nitrogen Input

**FIGURE 7:**
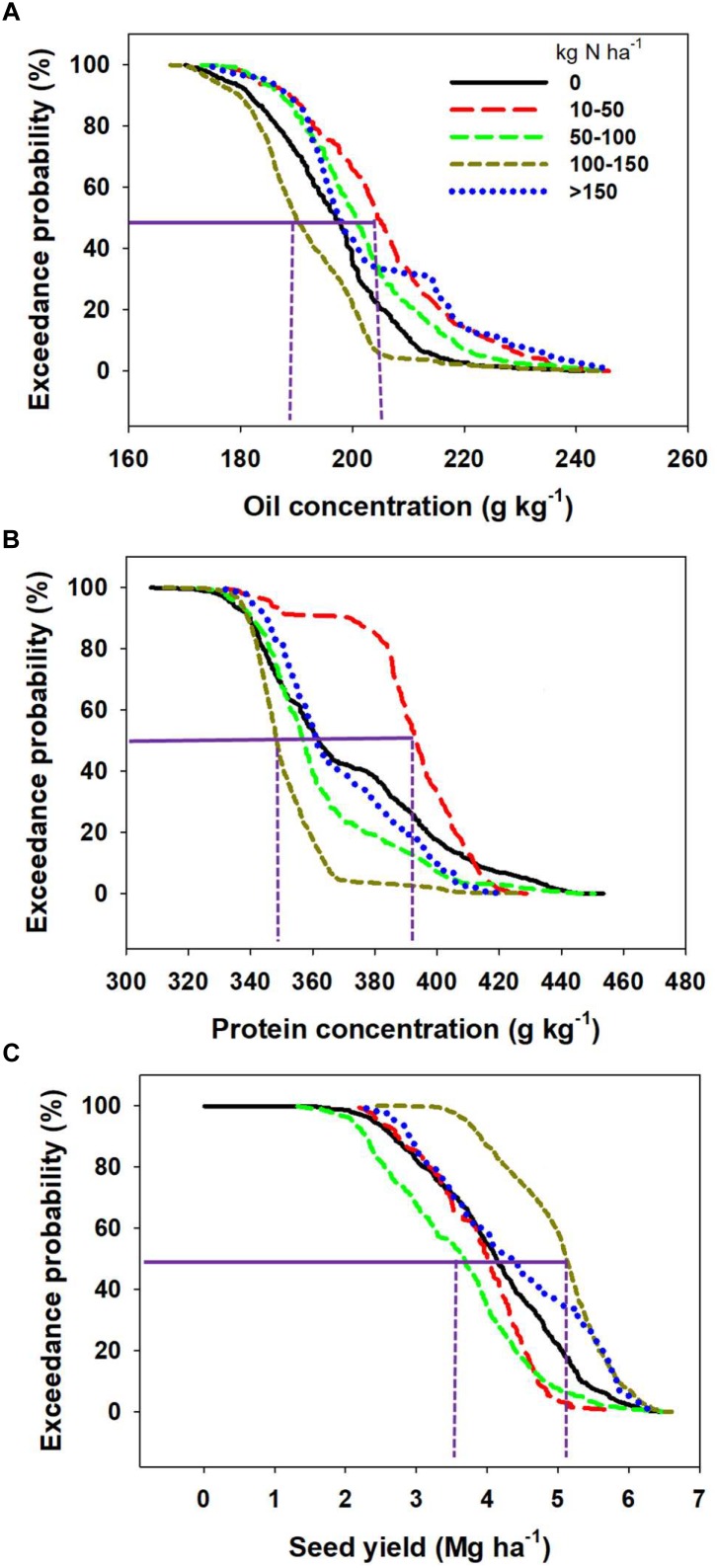
Exceedance probabilities for oil concentration **(A)**, protein concentration **(B)**, and seed yield **(C)** of soybean at different N rate. Exceedance probability here is defined as the probability (indicated in the *y*-axis) of obtaining oil, protein, or yield exceeding the indicated value (in the *x*-axis) for each fertilizer N rate category.

Oil and protein concentrations responded differently to fertilizer N input relative to seed yield (Figure 7). Exceedance probability calculates the probability of obtaining oil, protein, or yield exceeding a value for those parameters at each N rate category. The exceedance probability for each level of oil concentration increases when N inputs increased from 0 to 10–50 kg ha^-1^ but decreased as N fertilization increases greater than 50 kg ha^-1^ (Figure 7A). Therefore, there was a higher probability of obtaining greater oil concentration with N application ranging from 10-to-50 kg N ha^-1^ than when N fertilization is above 50 kg N ha^-1^. For example, there was a 20% exceedance probability of obtaining more than 200 mg kg^-1^ oil concentration with the fertilizer N range 100–150 kg N ha^-1^ but a 70% chance of getting similar oil values for the N fertilization range 10–50 kg N ha^-1^. Similar to oil concentration, the exceedance probability for protein concentration widens when N application increased from 0 to 10–50 kg N ha^-1^ and decreased substantially as N application increased above 10–50 kg N ha^-1^ (Figure 7B). It is worth highlighting that the exceedance probability when zero-N was applied is somewhat similar at the 50% probability level to the model when more than 150 kg N ha^-1^ is applied to soybeans, obtained both a probability for mean protein concentration slightly above of 36% (Figure 7B). The exceedance probability for seed yield, on the other hand, did not significantly change when N input increased from 0 to 100 kg N ha^-1^, but expanded when fertilizer N application increased from the 0 to 100 kg N ha^-1^ to the 100–150 kg N ha^-1^ range (Figure 7C).

A recent synthesis analysis on the impact of the rate, timing, and source of N fertilizer applied to soybean found that these factors contributed to less than one percent of the variation in seed yield (Mourtzinis et al., 2018). The yield benefits of smaller amounts of fertilizer as a starter (Osborne and Riedell, 2006; Gai et al., 2017) or full-N late application at R3–R4 stage (Ortez et al., 2018) were reported in the scientific literature. A positive impact of application of fertilizer on yield and seed composition, mainly in a high yielding E was reported by La Menza et al. (2017). Ray et al. (2006) reported a positive impact of application of higher rates of N on seed, protein, and oil yield but a decrease in protein concentration and protein to oil ratio. In a meta-analysis, Rotundo and Westgate (2009) reported a positive effect of N additions on seed protein concentration and more importantly on protein content (mg seed^-1^) for soybean. In a recently published study, Ortez et al. (2018) indicated that for soybean yield response to N fertilization is not strictly dependent on the yield E, but other factors influencing soil N supply and N fixation interaction. The above cited results have mixed message regarding impact of N application to seed yield and quality composition, demonstrating the complex influence of E (study) on the effect of this factor on the response variables. Probability of documenting yield gains with N fertilization will increase in Es where both N fixation and soil N supply are not capable of satisfying overall soybean plant N demand (e.g., Wilson et al., 2014; La Menza et al., 2017; Ortez et al., 2018).

### Genetics (Maturity Group)

**FIGURE 8:**
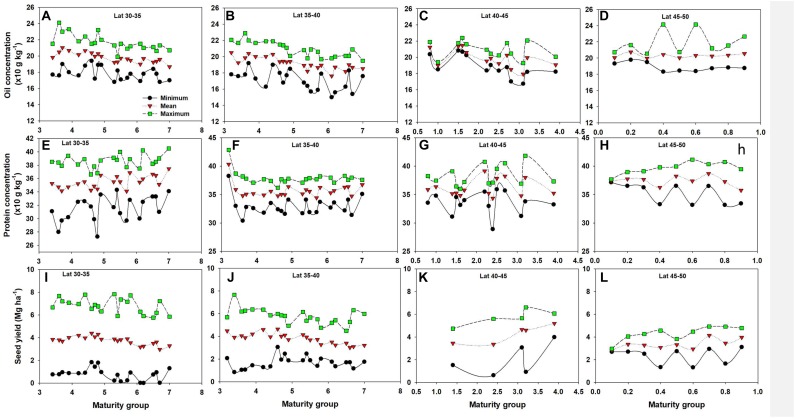
Minimum, mean, and maximum values of oil concentration **(A–D)**, protein concentration **(E–H)**, and seed yield **(I–L)** by latitude and different varieties maturity groups (MG).

Based on their photoperiod (day length) requirement soybean varieties are subdivided into different MGs. In southern latitudes (30–40 N), oil concentration tended to slightly decline as soybean MG increased, with protein portraying an opposite trend, primarily in the 30–35 N latitude, even though both were not statistically significant trends (Figure 8). There was also no significant difference among MGs in seed yield across all latitude groups. Evidence of genetic variability in soybean seed composition and yield is plentiful (TeKrony et al., 1984; Bajaj et al., 2008; Mourtzinis et al., 2017a,b). Our literature review on the effect of MG on oil and protein concentration found less published research, and few of the results presented in the scientific literature suggest a minor impact of MG on oil and protein relative to M such as planting date (Mourtzinis et al., 2017a).

In summary, E (e.g., temperature, solar radiation, precipitation) (Carrera et al., 2011; Rotundo et al., 2016) produced a significant impact on soybean seed yield and quality. M factors such as crop rotation and planting date had consistent positive or negative relation, whereas N application and other M factors have mixed effect one for seed composition and different for seed yield (Rotundo and Westgate, 2009; Bellaloui et al., 2015; Ortez et al., 2018). Other have reported the impact of G (e.g., variety, MG, plant traits) (Dardanelli et al., 2006; Bellaloui et al., 2009), in interaction with the E but this study only focused on main effects of E, M, and G. A detailed review chapter published by Bellaloui et al. (2011) provided a synthesis on the effects of G × E × M on soybean seed composition. The same authors concluded that a main scientific research gaps is related to studying the physiological mechanisms related to the variation on seed composition and the G × E × M interaction.

## Conclusion

This study provides a comprehensive analysis of G, M, and E factors influencing soybean yield quantity and quality across the United States Corn Belt. Because of the geographic coverage and numerous data points, it can serve as a baseline upon which future studies can design improved practices or measure future improvements in yields and quality. Multiple factors affect soybean seed composition and yield. E is a dominant factor for the significant variability in seed composition and yield (*R*^2^ > 70%). Among the impacts of crop M factors are: (i) negative effect of late planting date on oil concentration and yield in northern latitude (40–45 N); (ii) positive impact of crop rotation for both seed composition and yield; and (iii) mixed impacts of some M factors such as no-till, seed treatment, foliar nutrient and fungicide applications on seed composition. Application of N in smaller amount (less than 50 kg N ha^-1^) improved seed composition but seed yield was improved when N applications were above 100 kg N ha^-1^. MG differences in seed composition were not significant, but declining trend in oil and an increase in protein concentrations with increasing soybean MG were observed in southern latitudes (30–35 N). Exploring the G × E × M interaction is critical to better understand the current research gap and to move forward our science on this topic.

## Data Availability

All datasets generated for this study are included in the manuscript and/or the supplementary files.

## Author Contributions

YA, IC, and DD contributed to conception or design of the work. LP, MS, SN, SC, PK, SA, ML, FB, HK, LL, JG, SC, CS, JO, BG, GK, MS, KT, and RL collected the data. YA and IC contributed in data analysis and interpretation. YA, IC, LP, HK, SN, and CS drafted the article. LP, MS, SN, SC, PK, SA, ML, FB, HK, LL, JG, SC, CS, JO, BG, GK, MS, KT, RL, and IC contributed in critical revision of the article. IC gave final approval of the version to be published.

## Conflict of Interest Statement

The authors declare that the research was conducted in the absence of any commercial or financial relationships that could be construed as a potential conflict of interest.

## Abbreviations

DOY: day of the year
E: environment
G: genetics
M: management
MG: maturity group
